# Draft Genome Sequence of *Cronobacter sakazakii* Clonal Complex 45 Strain HPB5174, Isolated from a Powdered Infant Formula Facility in Ireland

**DOI:** 10.1128/genomeA.00778-14

**Published:** 2014-08-07

**Authors:** Arthur W. Pightling, Franco Pagotto

**Affiliations:** Listeriosis Reference Service, Research Division, Bureau of Microbial Hazards, Food Directorate, Health Products and Food Branch, Health Canada, Ottawa, Ontario, Canada

## Abstract

*Cronobacter sakazakii* is a food-borne pathogenic bacterium that may cause severe illness in neonates and the elderly. We present the genome sequence of a rare strain (ST40, CC45), commonly found in multiple food processing facilities and in powdered infant formula and only indicted in a single clinical case.

## GENOME ANNOUNCEMENT

*Cronobacter sakazakii* (synonym, *Enterobacter sakazakii*) is a food-borne pathogenic bacterium that causes outbreaks and sporadic cases of severe (albeit rare) meningitis, most often in neonates when contaminated powdered infant formula is ingested (1, 2). Multi-locus sequence typing of *C. sakazakii* isolates has revealed that some strains are more virulent than others and at least one group (ST4) is strongly associated with neonatal infections, while other sequence types have not been shown to be implicated in infections (3). Still, little is known about *C. sakazakii* virulence traits (4). Here, we present a high-quality draft genome sequence of a currently unrepresented group of *C. sakazakii* (ST40, CC45). Despite the presence of multiple ST40/CC45 isolates in our collection, obtained from either powdered infant formula or facilities making formula, none in our collection have been connected to any clinical cases and a search reveals that it has been indicted in only one clinical case occurring in China (http://pubmlst.org/perl/bigsdb/bigsdb.pl?page=info&db=pubmlst_cronobacter_isolates&id=486). Thus, this strain may be avirulent or only mildly virulent and may provide a useful outgroup for the identification of virulence factors.

*In silico* analysis of the multi-locus sequence typing (5) targets (*atpD*-3, *fusA*-15, *glnS*-28, *gltB*-22, *gyrB*-5, *infB*-38 and *pps*-19 (6)) indicates that strain HPB5174 belongs to the sequence-type 40 group and clonal complex 45 (3, 7). This finding is supported by analysis with pulsed-field gel electrophoresis, which yielded extremely rare patterns with both the XbaI and SpeI restriction endonucleases (BOM_CSXA1.0108 and BOM_CSSE1.0033). During a search of the *Cronobacter* PubMLST website (http://pubmlst.org/perl/bigsdb/bigsdb.pl?db=pubmlst_cronobacter_isolates) we found ten other isolates designated ST40/CC45 from India, United Kingdom, Australia, Netherlands, China, and Slovakia.

We generated short-read sequence data by preparing a paired-end library with the Nextera XT DNA sample preparation kit (Illumina, San Diego, CA) and reading the sequences on a MiSeq Benchtop Sequencer (Illumina) for 600 cycles, yielding 1,835,160 reads with an average length of 250.93 bases. Error correction was performed with BayesHammer (8) and reads were assembled *de novo* into a high-quality draft genome with SPAdes v3.0.0 (9), utilizing the MismatchCorrector tool. The assembly yielded 84 non-overlapping contiguous sequences (contigs) with a total length of 4,444,610 bases, 56.86% G+C content, and 103.61-fold sequencing coverage. The largest contig is 1,457,596 nucleotides in length and the weighted mean contig length (*N*_50_) is 526,913 nucleotides. Gene predictions and annotations were performed with the National Center for Biotechnology Information (NCBI) Prokaryotic Genome Annotation Pipeline (PGAP) (10). A total of 4,136 genes were identified, including 3,993 protein-coding regions, 39 pseudogenes, 2 clustered regularly interspaced short palindromic repeat (CRISPR) arrays, 23 ribosomal RNA, 76 transfer RNA, and 5 non-coding RNA sequences.

### Nucleotide sequence accession numbers.

This whole-genome shotgun project has been deposited at DDBJ/EMBL/GenBank under the accession number JNBN00000000. The version described in this paper is the first version, JNBN01000000.
